# Prospective assessment of AR splice variant and PSMA detection on circulating tumor cells of mCRPC patients: preliminary analysis of patients enrolled in PRIMERA trial (NCT04188275)

**DOI:** 10.1007/s10585-021-10118-7

**Published:** 2021-08-19

**Authors:** G. Francolini, M. Loi, V. Salvestrini, M. Mangoni, B. Detti, V. Di Cataldo, M. Aquilano, P. Pinzani, F. Salvianti, I. Desideri, M. Mariotti, P. Garlatti, G. Stocchi, L. P. Ciccone, S. Lucidi, G. Salvatore, M. Sottili, I. Meattini, L. Livi

**Affiliations:** 1 Department of Biomedical, Experimental and Clinical Sciences “M. Serio”, University of Florence, Florence, Italy; 2 Radiation Oncology Unit, Oncology Department, Azienda Ospedaliero Universitaria Careggi, Largo G. A. Brambilla 3, 50134 Florence, Italy

**Keywords:** Metastatic prostate cancer, CTCs, PSMA, AR splice variants

## Abstract

In our institution, a prospective observational trial testing micro-RNA (miRNA) and ARV7 mutational status in metastatic, castration resistant prostate cancer (mCRPC), is currently recruiting (PRIMERA trial, NCT04188275). A pre-planned interim analysis was performed when 50% of the planned accrual was reached. In this report, we explored the predictive value of Circulating Tumor Cell (CTC) detection in mCRPC patients undergoing 1st line therapy. Moreover, ARV7, ARFL, PSMA and PSA expression on CTC was reported to explore potential correlation with patient prognosis and response to therapy. PRIMERA is a prospective observational trial enrolling mCRPC patients undergoing standard treatment (ARTA + ADT) after I line ADT failure. Clinical and pathological features were collected. Outcomes selected for this preliminary analysis were time to castration resistance (TTCR), PSA at 8 weeks after ARTA therapy start, PSA drop at 8 weeks, Overall PSA drop, PSA nadir. Correlation between these outcomes and CTC detection was tested. Expression of ARV7, ARFL, PSA and PSMA was explored in CTC+ patients to assess their prevalence in this cohort and their impact on selected outcomes. Median TTCR was significantly shorter in CTC+ vs CTC− patients (32.3 vs 75 months, respectively, p = 0.03) and in ARFL+ vs ARFL− patients (30.2 vs 51.1 months, respectively, p = 0.02). ARV7, PSMA and PSA expression on CTC had no impact on median TTCR, nor on biochemical response to therapy. Patients in whom CTC and ARFL expression were detected had significant reduced TTCR. However, PSA response was not influenced by CTCs detection and specific biomarkers expression.

## Background

Androgen deprivation therapy (ADT) represent the cornerstone for treatment of metastatic prostate cancer. However, vast majority of patients eventually progresses, despite ADT, into metastatic castration resistant prostate cancer (mCRPC) status. Up to 2004, no standard therapy able to improve prognosis of these patients was available [1]. COU-AA 302 and PREVAIL trials demonstrated the benefit in terms of Overall Survival (OS) in chemotherapy-naive patients treated with Abiraterone and Enzalutamide if compared to placebo, respectively, paving the way to the use of Androgen receptor targeted agents (ARTA) in the mCRPC setting [2, 3]. Radiopharmaceuticals represent another available treatment option for these patients; Alsympca trial results showed that Radium-223 bone-targeted therapy yielded significant OS benefit [4] and recent data from literature suggest promising response rate after treatment with Lutethium-PSMA [5]. However, no clear recommentation can be made about the most effective 1st line treatment for mCRPC, and a univocal treatment algorithm is not currently available [6]. Many efforts have been done to identify predictive biomarkers and tailor treatment strategy for selected patients. Moreover, biomarker identification could help to understand disease behavior in metastatic hormone sensitive prostate cancer (mHSPC) as well. Detection of Androgen receptor splice variant-7 (ARV7) on circulating tumor cells (CTCs) has been shown to significantly predict shorter progression free survival (PFS) and overall survival (OS) after ARTA treatment, suggesting that alternative options should be offered in these patients [7]. Full‐length androgen receptor (ARFL) expression may further help to predict response and survival after ARTA therapy [8]. Prostate specific membrane antigen (PSMA) is a type II membrane glycoprotein encoded in the folate hydrolase 1 (FOLH1) gene [9]. PSMA expression is important both for its diagnostic and therapeutic implications [10]. In our institution, a prospective observational trial testing micro-RNA (miRNA) and ARV7 mutational status in mCRPC is currently recruiting (PRIMERA trial, NCT04188275) [11]. A pre-planned interim analysis was performed when 50% of the planned accrual was reached.

## Objective

In this report, we explored the predictive value of CTC detection in mCRPC patients undergoing I line ARTA therapy. Moreover, ARV7, ARFL, PSMA and Prostate Specific Antigen (PSA) expression on CTC was reported to explore potential correlation with patient prognosis and response to therapy.

## Design, setting, and participants

### Population

PRIMERA is a prospective, observational trial enrolling mCRPC patients undergoing standard treatment (ARTA + ADT) after I line ADT failure. CRPC was defined according to European association of Urology guidelines [6] and patients were treated with either Abiraterone or Enzalutamide according to clinical choice. Previous chemotherapy administration constituted an exclusion criterion. All patients signed informed consent, protocol was approved by local ethical committee and registered on Clinicaltrials.gov (NCT04188275).

### CTC enrichment and analysis

Blood samples for CTC detection were repeated at treatment start, 8 weeks from treatment start and at disease progression. AdnaTest ProstateCancerPanel AR-V7 (Qiagen Gmbh, Hilden, Germany) was used for CTC enrichment and characterisation. 10 ml of blood were collected before starting a new line of therapy into collection tubes BD vacutainer glass ACD solution B (Becton Dickinson, Franklin Lakes, New Jersey, USA). CTCs were isolated by immuno- magnetic beads recognizing epithelial and tumor-associated antigens (AdnaTest Prostate Cancer Select). Cell lysis and reverse transcription were performed according to the manufacturer’s instructions. mRNA was obtained and reverse-transcribed using the AdnaTest Prostate Cancer Panel ARV7 and SensiScript RT kits (Qiagen, Hilden, Germany). We evaluated the expressions of PSA, PSMA, AR and ARV7, using Reverse Transcription–quantitative real-time PCR (RT-qPCR). Housekeeping gene (CD45 and GAPDH) expression was used to assess the success of the experimental protocol for CTC enrichment, mRNA isolation and gene expression analysis. A sample was considered positive—indicating the presence of CTCs—if at least one prostate cancer-associated transcript (PSA, PSMA, AR or ARV7) was detected.

### Outcome measurements and statistical analysis

Clinical and pathological features were collected. Outcomes selected for this pre-planned preliminary analysis were time to castration resistance (TTCR, defined as time between ADT start and CRPC occurrence), PSA at 8 weeks after ARTA therapy start, PSA drop at 8 weeks (defined as difference between PSA at 8 weeks after ARTA therapy start and baseline PSA), Overall PSA drop (defined as difference between last PSA registered and baseline PSA), PSA nadir (defined as PSA lowest value registered during ARTA therapy). Descriptive analysis was performed to summarize patient- and CTC-related characteristics in the study population. Correlation between these outcomes and CTC detection was tested. Furthermore, expression of ARV7, ARFL, PSA and PSMA was explored in CTC+ patients to assess their prevalence in this cohort and their impact on selected outcomes. Chi-square test was performed to test the association between ARV7, ARFL, PSA and PSMA expression. Kaplan–Meier analysis was performed to assess the correlation of outcomes with CTC detection and expression of ARV7, ARFL, PSA and PSMA. All statistical analyses were performed with MedCalc version 18.9.

## Results and limitations

### Overall cohort and detection rate

Overall, 28 patients were included in the present cohort. Of these, CTCs were detected at treatment start in 15 patients (53.6%). Out of the 15 patients in whom CTC were detected (CTC+), 2(13.3%), 9(60%) 12(80%) and 11(73.3%) patients expressed ARV7, ARFL, PSA and PSMA, respectively. Principal characteristics and treatment outcomes measured in the overall population are summarized in Table 1.

**Table 1:** Summary of principal characteristics and treatment outcomes measured in the overall population

Baseline gleason score	< 7: 2 (7.2%) 7: 13 (46.4%) > 7: 13 (46.4%)
Median time to castration resistance (months)	48
Median PSA at castration resistance occurrence (ng/ml)	7.92
mCRPC therapy	Enzalutamide: 13 (46.4%) Abiraterone: 15 (53.6%)
Median PSA at 8 weeks (ng/ml)	2.8
Median PSA drop at 8 weeks (ng/ml)	− 3.5
Median overall PSA drop (ng/ml)	− 5.5
Median PSA nadir (ng/ml)	1.03

### *AR, PSA and PSMA expression in CTC* + *patients*

**Fig. 1:**
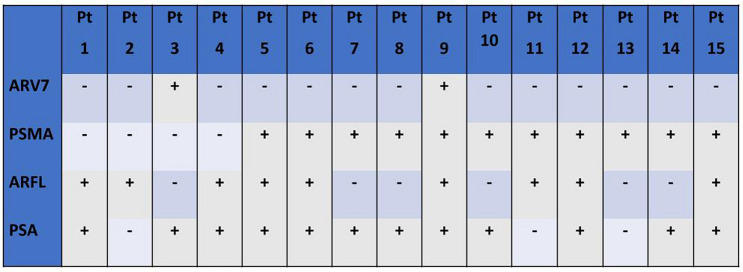
Expression of ARV7, PSMA, ARFL and PSA in all CTC positive patients (pt 1–15)

Expression of ARV7, PSMA, ARFL and PSA in the 15 CTC positive patients is summarized in Fig. 1. Chi square test showed no difference in terms of ARFL, PSA and PSMA expression between ARV7+ and ARV7− CTCs (p = 0.76, p = 0.46 and 0.43, respectively). Moreover, ARFL, PSA and ARV7 expression did not significantly differ between PSMA+ and PSMA− CTCs (p = 0.48, p = 0.77 and 0.58, respectively). ARFL, PSMA and ARV7 were equally expressed in PSA+ and PSA− CTCs (p = 0.71, 0.77 and 0.46, respectively). Expression of PSA, PSMA and ARV7 was comparable between ARFL+ and ARFL− CTCs (p = 0.22, 0.48 and 0.76 respectively).

### Correlation between CTCs detection and selected outcomes

**Fig. 2:**
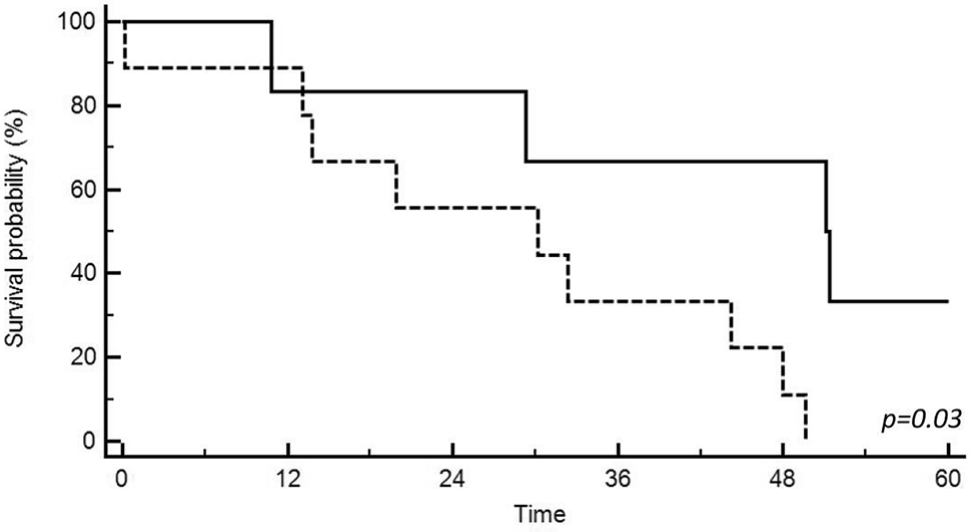
Time to castration resistance in CTC positive (solid line) vs CTC negative (dotted line) patients

Median TTCR was significantly shorter in CTC+ vs CTC− patients (32.3 vs 75 months, respectively, p = 0.03). Kaplan–Meier analysis for TTCR in CTC positive vs CTC negative patients is reported in Fig. 2. However, no difference in terms of biochemical response to ARTA therapy was found between CTC+ and CTC− patients. Indeed, median values of PSA at 8 weeks (3.1 vs 2.5 ng/ml, p = 0.8), PSA drop at 8 weeks (− 6.3 vs − 2.4 ng/ml, p = 0.47), Overall PSA drop (− 18.5 vs − 3.4 ng/ml, p = 0.17) and PSA nadir (1.1 vs − 0.6 ng/ml, p = 0.48) did not differ between CTC+ and CTC− patients. Treatment outcomes in the Circulating Tumor Cell (CTC) positive vs negative population are summarized in Table 2.

**Table 2:** Summary of treatment outcomes in the circulating tumor cell (CTC) positive vs negative population

Measured outcome	Results	p
Median Time to castration resistance (months)	CTC+: 32.3 (95% CI 19.8; 49.6)	**0.007**
	CTC−: 75.4 (95% CI 41.4; 99.3)	
Median PSA at 8 weeks (ng/ml)	CTC+: 3.1 (95% CI 0.68; 10.5)	0.8
	CTC−: 2.5 (95% CI 0.8; 8.9)	
Median PSA drop at 8 weeks (ng/ml)	CTC+: − 6.3 (95% CI − 30.7; 0.34)	0.47
	CTC−: − 2.4 (95% CI − 7.9; 0.3)	
Median Overall PSA drop (ng/ml)	CTC+: − 18.6 (95% CI − 43; − 3.5)	0.17
	CTC−: − 3.4 (95% CI − 14.8; − 1.5)	
Median PSA nadir (ng/ml)	CTC+: 1,1 (95% CI 0.28; 3.8)	0.48
	CTC−: − 0,64 (95% CI 0.19; 1.7)	

Bold indicate statistically significant value

### *Correlation between AR, PSA and PSMA expression in CTC* + *patients and selected outcomes*

**Fig. 3:**
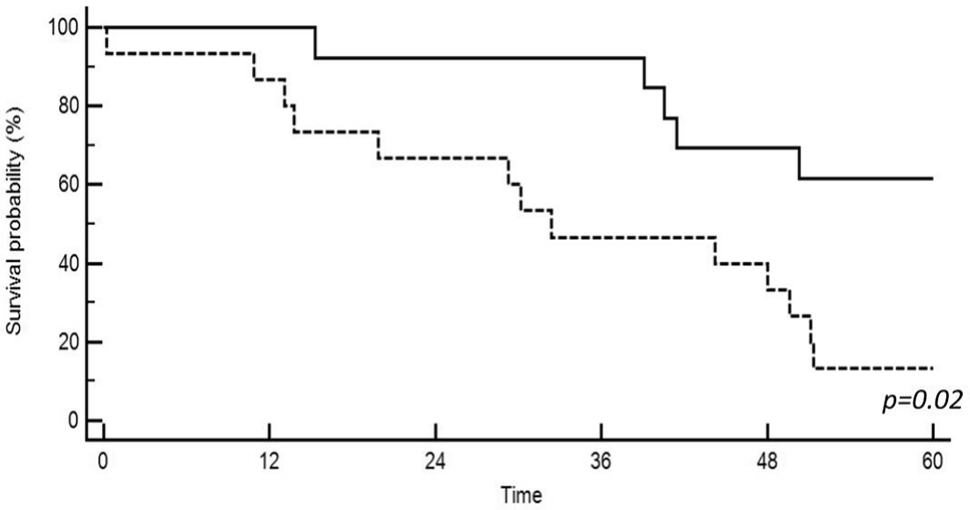
Time to castration resistance in ARFL positive (solid line) vs ARFL negative (dotted line) patients

ARV7 expression on CTCs had no impact on median TTCR (30.1 vs 32.3 months in ARV7+ vs ARV7− patients, respectively, p = 0.19), neither on biochemical response to ARTA therapy, with median PSA at 8 weeks, PSA drop at 8 weeks, overall PSA drop and PSA nadir of 6.1 vs 3.1 ng/ml (p = 0.73), − 25.4 vs − 3.7 ng/ml (p = 0.39), − 28 vs − 6.3 ng/ml (p = 0.61) and 3.6 vs 1.1 ng/ml (p = 0.86), respectively. Significant impact of ARFL expression was detected in terms of TTCR, with 30.2 vs 51.1 months (p = 0.02) in ARFL+ vs ARFL− patients, respectively. Kaplan–Meier analysis for TTCR in ARFL+ vs ARFL− patients is reported in Fig. 3. However, no influence of ARFL expression was detected in terms of biochemical response to ARTA therapy, with median PSA at 8 weeks, PSA drop at 8 weeks, overall PSA drop and PSA nadir of 1.1 vs 3.3 ng/ml (p = 0.7), − 18.5 vs − 1.3 ng/ml (p = 0.19), − 18.6 vs − 12.9 ng/ml (p = 0.4) and 1.1 vs 2.01 ng/ml (p = 0.63), respectively. Expression of PSMA on CTCs had no significant impact on TTCR (44.2 vs 13.8 months, respectively, p = 0.96), and biochemical response to therapy was comparable between PSMA+ and PSMA− patients, with median PSA at 8 weeks, PSA drop at 8 weeks, overall PSA drop and PSA nadir of 1.1 vs 9.2 ng/ml (p = 0.2), − 6.3 vs − 18 ng/ml (p = 0.51), − 18.6 vs − 21 ng/ml (p = 0.6) and 0.9 vs 4.4 ng/ml (p = 0.51), respectively. No significant impact of PSA expression on CTCs was noticed in terms of TTCR (44.2 vs 29.3 months in PSA + vs PSA − patients, respectively, p = 0.21). Moreover, no impact of PSA expression on biochemical outcomes was found, with median PSA at 8 weeks, PSA drop at 8 weeks, overall PSA drop and PSA nadir of 3.3 vs 1 ng/ml (p = 0.66), − 12.4 vs − 1.7 ng/ml (p = 0.66), − 20.5 vs − 4.7 ng/ml (p = 0.4) and 1.9 vs 0.3 ng/ml (p = 0.38), respectively. A summary of treatment outcomes measured in the Circulating Tumor Cell (CTC) positive population, divided for ARV7, PSMA, ARFL and PSA status, is summarized in Table 3.

**Table 3:** Summary of treatment outcomes measured in the circulating tumor cell (CTC) positive population, divided for ARV7, PSMA, ARFL and PSA status

Measured outcome	Status (n)	Results (95% CI)	p
Median time to castration resistance (months)	ARV7+	30.1 (30.1; 90.5)	0.19
	ARV7−	32.3 (13.8; 90.5)	
	PSMA+	44.2 (29.3; 51.1)	0.96
	PSMA−	13.8 (0.17;90.5)	
	ARFL+	30.2 (13.8; 44.2)	**0.02**
	ARFL−	51.1 (29.3; 77.2)	
	PSA+	44.2 (13.8; 51.1)	0.21
	PSA−	29.3 (19.8; 32.3)	
Median PSA at 8 weeks (ng/ml)	ARV7+	6.1 (n/a)	0.73
	ARV7−	3.1 (0.78; 8.99)	
	PSMA+	1.1 (0.51; 5.45)	0.24
	PSMA−	9.27 (n/a)	
	ARFL+	1.1 (0.36; 11.29)	0.72
	ARFL−	3.33(0.29; 319.9)	
	PSA+	3.3 (0.39; 12.25)	0.66
	PSA−	1 (n/a)	
Median PSA drop at 8 weeks (ng/ml)	ARV7+	− 25.4 (n/a)	0.39
	ARV7−	− 3.7 (− 34.7; 0.05)	
	PSMA+	− 6.3 (− 29.3; 0.28)	0.51
	PSMA−	− 18 (n/a)	
	ARFL+	− 18.5 (− 243.8; − 0.63)	0.19
	ARFL−	− 1.3 (− 30.5; 52.4)	
	PSA+	− 12.4 (− 42.5; 0.06)	0.66
	PSA−	− 1.7 (n/a)	
Median overall PSA drop (ng/ml)	ARV7+	− 28 (n/a)	0.61
	ARV7−	− 6,3 (− 45.37; − 2.74)	
	PSMA+	− 18.6 (− 45.1; − 1.6)	0.6
	PSMA−	− 21 (− 157.5; − 4.35)	
	ARFL+	− 18.6 (− 245.6; − 4.07)	0.4
	ARFL−	− 12.9 (− 43.5; 0.3)	
	PSA+	− 20.52 (− 45.6; 3.5)	0.47
	PSA−	− 4.74 (n/a)	
Median PSA nadir (ng/ml)	ARV7+	3.6 (n/a)	0.86
	ARV7−	1.1 (0.3; 3.7)	
	PSMA+	4.4 (n/a)	0.51
	PSMA−	0.9 (0.24; 3.5)	
	ARFL+	1.1 (0.07; 3.8)	0.63
	ARFL−	2.01 (0.23; 230.27)	
	PSA+	1.9 (0.3; 6.7)	0.38
	PSA−	0.3 (n/a)	

Bold indicate statistically significant value

## Discussion

The present analysis represents a helpful snapshot of a prospectively enrolled cohort of mCRPC patients undergoing I line ARTA therapy. Overall, our results showed a CTC detection rate in the present series of 53.6%, inferior to what previously reported in the literature [12–14] but comparable to the results of a recent study exploiting the same CTC enrichment method [15]. In this regard, lack of standardization among the high number of different CTC detection approaches and the different clinical characteristics of the patients’ cohorts must be taken into account when considering the reported results. On the other hand, our results confirm CTCs as a prognostic biomarker in metastatic prostate cancer. Indeed, the detection of CTCs was related to shorter TTCR, suggesting that this marker could be related to higher subclinical burden of disease and increased potential to overcome ADT in hormone sensitive status. This is in line with previous results from literature; results from SWOG S1216 trial showed that Baseline CTC detection in mHSPC was associated with higher prostate-specific antigen (PSA), extensive disease, and bony metastasis [16]. Moreover, Li et al. recently showed in a multicenter prospective cohort study that AR-V7 expression in primary cancer tissue is correlated with poor prognosis for mHSPC patients receiving ADT, confirming the role of this biomarker in an earlier scenario [17]. Our data did not confirm impact of CTC detection on biochemical response to I line ARTA therapy. Conversely, more mature data from SWOG S1216, presented at ASCO 2020, pointed out the relationship between baseline CTC count, PSA response and PFS [18]. However, patients enrolled in SWOG S1216 trial were tested in mHSPC status, while PRIMERA patients were already progressed to mCRPC, suggesting the pivotal role of CTCs in predicting the early outcome of hormone sensitive disease rather than response to therapy when CRPC already occurred. Moreover, to further explain these discrepancies, it has to be taken into account that we used an indirect method for CTC detection, not allowing CTC imaging and count, but based on the detection of tumor-specific transcripts on CTCs. Due to CTC heterogeneity, the expression levels of the mRNA markers under investigation are not necessarily correlated to the number of tumor cells in the circulation. Early prognostic role of CTCs detection could be helpful to determine mHSPC patients in whom treatment intensification could yield significant benefit, despite clinical classifications proposed by current clinical trials exploring this issue [19–21].Our results did not show any correlation between ARV7, ARFL, PSMA and PSA cell expression on CTCs. Previous data from literature showed significantly higher ARFL expression in AR-V7-positive patients; however, Del re et al. analyzed mRNA isolated from exosomes, rather than CTCs, conversely from the present analysis [8]. Nevertheless, a CTC analysis conducted on patients enrolled in the SAKK 08/14 IMPROVE trial showed that comparable rates of ARFL expression was detected in ARV7+ and ARV7− patients, consistently with present results [22]. Interestingly, no relationship between ARV7 and PSMA expression was found. Role of PSMA-PET imaging and Lutethium-PSMA therapy in advanced ARV7 negative mCRPC was previously questioned, due to the potential correlation between absence of ARV7 and lack of PSMA expression [23]. Indeed, ADT is normally considered to upregulate PSMA expression [24], and also ARTA showed to have similar influence both in castration sensitive and castration resistant cellular models [25]. However this link between AR suppression and PSMA upregulation may be reduced by presence of AR splice variants (i.e. ARV7) [26]. Nonetheless, present data suggest that PSMA expression could be detected also despite ARV7 absence, and that PSMA based imaging and radiomethabolic therapy may be clinically helpful also in these patients. Despite previous reports indicating a prognostic role of AR-V7 and PSMA expression on clinical outcomes [8] our data did not suggest any significant impact of these CTC molecular features on biochemical outcomes. Indeed, AR splice variants showed to have significant impact on observed benefit after systemic therapies in mCRPC setting, suggesting that response to ARTA, but not to taxane chemotherapy, may be negatively affected by ARV7 expression [7]. Understanding the role of ARFL is more complex, because of its highly heterogeneous expression [27]. Moreover, some authors demonstrated that AR amplification was associated to improved response to ARTA [28] while others observed poor response to enzalutamide and abiraterone associated to this feature [8]. Data from the present analysis suggest that ARFL expression is related to significant reduction in terms of TTCR, underlining the negative prognostic factor of this feature. Nonetheless, no impact of ARFL expression was detected on biochemical outcomes after ARTA therapy. However, it should be noted that survival data from the current series are not mature yet, considering that only two patients from the overall cohort progressed under I line ARTA therapy, and that the role of biochemical outcome as a surrogate endpoint is not universally recognized. Moreover, the role and activity of AR could be influenced by their localization (transcriptionally inactive in the cytoplasm while active when localized in the nucleus) [29, 30]. Therefore, cytoplasmic rather than surface expression of these markers could predict response to therapy, and differences in terms of their impact on response to therapy may be related to different site of localization of these proteins within CTCs, and that could be investigated using a different methodological approach. Biomarker analysis may be helpful in particular scenarios. For example, CTC molecular profiling may help to identify patients with oligometastatic disease who may benefit from metastasis directed therapy. Results from ARTO Trial (NCT NCT03449719) may help to explore this issue [31]. Of note, blood samples for CTC detection were repeated at 8 weeks from treatment start and at disease progression, but results were not mature yet and were not included in the present analysis. Longitudinal biomarker assessment may further help to explore correlation between CTCs detection and response to ARTA therapy. The present study provides data about detection rate and molecular profiling of CTCs in an homogeneous cohort of I line mCRPC patients treated with ARTA, prospectively enrolled within an observational trial. Limitation of this preliminary analysis is the low number of patients at current stage of advancement of the trial, which may reduce its statistical reliability. Early outcomes need to be validated with final analysis on overall cohort. Moreover, TTCR is a questionable endpoint considering that all patients were enrolled at I line mCRPC treatment start. However, considering the non-interventional nature of the trial, TTCR and its relation with biomarker analysis could in any case be of interest in this setting. Moreover, ARTA therapy has been shown to confer significant benefit in mHSPC as well [32], and biomarker analysis related to earlier outcomes in this population are explorative for use of these therapies in earlier scenarios.

## Conclusions

We presented the preliminary analysis of a prospective observational trial exploring the baseline prevalence of CTCs and their molecular profiling (ARV7, ARFL, PSA and PSMA expression) in a population of mCRPC patients undergoing I line ARTA therapy. Results suggested that patients in whom CTC were detected had significant reduced TTCR. Furthermore, CTC positive patients in whom ARFL expression was detected had significant reduction in TTCR. However, biochemical outcome was not influenced by CTCs detection and specific biomarkers expression.

## Data Availability

Research data are stored in an institutional repository and will be shared upon request to the corresponding author.
